# New breast cancer prognostic factors identified by computer-aided image analysis of HE stained histopathology images

**DOI:** 10.1038/srep10690

**Published:** 2015-05-29

**Authors:** Jia-Mei Chen, Ai-Ping Qu, Lin-Wei Wang, Jing-Ping Yuan, Fang Yang, Qing-Ming Xiang, Ninu Maskey, Gui-Fang Yang, Juan Liu, Yan Li

**Affiliations:** 1 Department of Oncology, Zhongnan Hospital of Wuhan University, Hubei Key Laboratory of Tumor Biological Behaviors & Hubei Cancer Clinical Study Center, Wuhan 430071, China; 2 Key State Laboratory of Software Engineering, School of Computer, Wuhan University, Wuhan 430072, China; 3 Department of Pathology, Zhongnan Hospital of Wuhan University, Wuhan 430071, China

## Abstract

Computer-aided image analysis (CAI) can help objectively quantify morphologic features of hematoxylin-eosin (HE) histopathology images and provide potentially useful prognostic information on breast cancer. We performed a CAI workflow on 1,150 HE images from 230 patients with invasive ductal carcinoma (IDC) of the breast. We used a pixel-wise support vector machine classifier for tumor nests (TNs)-stroma segmentation, and a marker-controlled watershed algorithm for nuclei segmentation. 730 morphologic parameters were extracted after segmentation, and 12 parameters identified by Kaplan-Meier analysis were significantly associated with 8-year disease free survival (*P* < 0.05 for all). Moreover, four image features including TNs feature (HR 1.327, 95%CI [1.001 - 1.759], *P* = 0.049), TNs cell nuclei feature (HR 0.729, 95%CI [0.537 - 0.989], *P* = 0.042), TNs cell density (HR 1.625, 95%CI [1.177 - 2.244], *P* = 0.003), and stromal cell structure feature (HR 1.596, 95%CI [1.142 - 2.229], *P* = 0.006) were identified by multivariate Cox proportional hazards model to be new independent prognostic factors. The results indicated that **C**AI can assist the pathologist in extracting prognostic information from HE histopathology images for IDC. The TNs feature, TNs cell nuclei feature, TNs cell density, and stromal cell structure feature could be new prognostic factors.

Breast cancer (BC) is the most common malignant tumor and the second leading cause of cancer death in women worldwide^1^, and its mortality is beginning to decrease owe to early detection and improved therapies^2^. Though adjuvant systemic therapies significantly improve patient survival, the therapeutic concept for BC has gradually shifted from “maximally tolerated treatment” to “minimally necessary treatment”^3^. For better individualized treatment, clinical management of BC relies on prognostic factors to accurately predict the risk of recurrence and metastasis after “clinically curative therapies” so as to avoid either over-treatment or under-treatment^4^.

A variety of clinical and pathological factors have been used to assess BC prognosis^5^. Combinations of those factors yield different predictive tools/models, such as tumor-node-metastasis (TNM) stage, Nottingham Prognosis Index (NPI)^6^,^7^(Figure S1). However, BC is a highly heterogeneous disease and traditional prognostic tools have failed to evaluate risk in individual patients, especially for the early BC patient^6^. Molecular techniques, such as gene expression profiling^8^, have the potential to provide valuable information beyond that obtained by traditional factors, but their role is limited by the universality of the technologies^9^. Moreover, the results of expensive molecular assays can be confounded by the admixture of normal breast tissue or inflammatory cells^9^. Thus, in current clinical practice, pathologists remain rely on relatively inexpensive and reproducible morphologic assays (HE and immunohistochemistry methods) to grade tumor and guide clinical decision^10^.

Grading of hematoxylin-eosin (HE) stained histopathology image is one of the standard practices for BC prognosis prediction^11^. Histological grade provides an inexpensive and routinely applicable view of the biological characteristics and clinical behaviors of BC. Currently, World Health Organization adopts the Nottingham grading system (NGS) to determine the tumor grade^12^. The NGS score is achieved by pathologists to qualitatively evaluate the tubule formation, nuclear pleomorphism, and mitotic count. Such qualitative work can lead to wide observer variability even among the specialized breast pathologists^13^. Furthermore, tumor microenvironment mediates the initiation and progression of BC^14^, and causes morphologic changes at the cellular and tissue levels which are visible in histopathology images. Nevertheless, the wealth of indispensable information within the tumor microenvironment is not reflected in the NGS and also lost in molecular tests^15^. For instance, even tumors in the same molecular classification may have different image-based features with significantly different survival outcomes^16^. Therefore, it is necessary to explore new pathological prognostic factors for BC based on analysis of *in situ* tumor information from HE histopathology images.

The advance of digital pathology and high-throughput technologies greatly facilitate the application of image analysis techniques in pathology^17^. Computer-aided image (CAI) analysis has great potential to overcome the inconsistence arise from subjective interpretation, and extract new information beyond conventional pathological parameters at the same time^18^,^19^,^20^. Quantitative analysis of HE images is an emerging field gaining more and more importance^20^.Various methods have been proposed for objects (gland, nuclei, and mitosis) segmentation^21^, malignant regions classification^22^, and computer-aid diagnosis, grade, and prognosis^23^,^24^.

Though the histologic type provides prognostic information, the majority type (60% - 75%) is invasive ductal carcinoma (IDC) of the breast; the role of traditional histologic typing in prognosis is limited^11^. Exploratory study suggests that cancer invasion is largely due to the collective behaviors of cancer cell groups, i.e. tumor nests (TNs), and in-depth study on the TNs features could reveal much richer useful information on tumor progression and prognosis^25^. To achieve this goal, a sound methodology should be established to define the TNs and to distinguish major features of TNs. In previous work^24^, we proposed an algorithm, which based on a pixel-wise support vector machine (SVM) classifier to segment TNs-stroma and a marker-controlled watershed to segment cell nuclei, to realize the automatic analysis of HE histopathological images from IDC.

In this work, we used the method proposed in Qu^24^ on 1,150 HE histopathology images from IDC patients. We extracted a rich set of quantitative morphological features from pixel-level, object-level and semantic-level information, and analyzed their correlations with 8-year disease free survival (8-DFS). The main steps of CAI proposed in this study are described in Fig. 1.

## Results

### Major clinical pathological characteristics of the patients

The main demographic, clinical and pathological characteristics of the 230 IDC patients are summarized in Table 1. The median age was 53 years (range, 27–85 years). The positive rates of estrogen receptor (ER) and human epidermal growth factor receptor 2 (HER2) were 45.7% and 36.1%, respectively. In terms of histological grade, 20 (8.7%) patients were classified as histological grade 1, 174 (75.7%) histological grade 2, and 36 (15.6%) histological grade 3. At the median follow-up of 105 months, among the 230 cases, 152 (66.1%) patients had tumor recurrence, and the median 8-DFS was 50.1 months (95% confidence interval [CI]:38.3 - 62.0 months) as analyzed by Kaplan-Meier survival curve.

### Image segmentation

**Figure 2:**
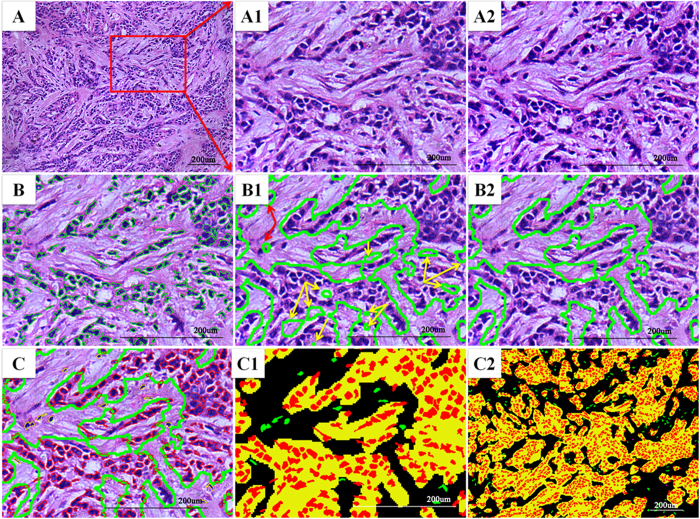
Overview of the results of CAI pipeline. (**A**) Original HE image. The other images are local amplification of red rectangle region in **A** (**A1**) Image before preprocessing. (**A2**) Preprocessing result shows improved image quality. (**B**) Nuclei segmentation result by marker-controlled watershed algorithm, the green rectangle region represents an individual nuclear region. (**B1**) Initial TNs-stroma segmentation result, the yellow arrows indicate the TNs regions which were incorrectly labeled as stroma, and red arrows indicate the stroma regions which were incorrectly labeled as TNs. (**B2**) Final segmentation result after postprocessing. (**C**) Integration of TNs-stroma and nuclei segmentation results. (**C1**, **C2**) All image objects were subclassified in the form of pseudocolor image after segmentation (epithelial region = yellow; stroma matrix = black; epithelial cell nuclei = red; stroma non-round cell nuclei = green; stroma round cell nuclei = purple). TNs: tumor nests

So far, the limiting factor for quantitative HE image analysis is the absence of a robust and accurate segmentation algorithm to distinguish objects (tumor nests, gland, nuclei etc.) of interest from the background. Apart from histological grade, there are many other morphologic features of BC that have been proposed as prognostic factors, including angiogenesis, lymphocytes infiltration, and tumor-associated inflammation. Segmentation of the tissue into different components is the first step toward automatic morphometry. We used a pixel-wise SVM classifier for tumor nests (TNs)-stroma segmentation and a marker- controlled watershed for nuclei segmentation. The segmentation results are presented in Fig. 2 in the form of pseudo-color images; all pixels were sub-classified into TNs (yellow), stroma (black), epithelial nuclei (red), stromal round nuclei (infiltrating immune cells, IICs in purple), and stromal non-round nuclei (cancer-associate fibroblastic cells [CAFs] and angiogenic vascular cells [AVCs] in green)^13^.

### Parameter extraction and dimensionality reduction

The morphologic characteristics of the tissue, cells, and nuclei are relatively complex. Some parameters could be easily described, for example, a tubule formation and high nuclear-to-cytoplasmic ratio, but most are difficult to describe, hard to learn, and often require long time before a pathologist can grasp. Image analyses emulate the expert learning to recognize objects in images; can transform microscopically-observed parameters from semi-quantitative values to quantitative data. The reproducible, objective morphologic results generated from image analysis provide means to encode image information into a set of discriminatory measurable values.

We extracted 730 image parameters (Table S1) from multiple classes of different components. These parameters could be divided into pixel-level parameters (n = 400) including intensity, color and texture variables; object-level parameters (n = 314) including morphometry (object size and shape etc.) and topological variables; and semantic-level parameters (n = 16) including nest area/stroma area ratio, stroma round cell density, and nuclei/cytoplasm ratio etc (Table S2). The pixel-level parameters were not considered in this work because they are the least interpretable in terms of current biological knowledge^18^. For the other two sets of parameters, we conducted univariate survival analysis for parameters dimensionality reduction to screen for clinically significant image parameters. This yielded 14 parameters for further analysis; 12 among these were significantly associated with 8-DFS (*P* < 0.05 for all) (Table 2 and Table 3).

### Clinical value of morphologic parameters for 8-DFS prediction

The object-level parameters refer to the properties (such as area, perimeter, and fractal dimensions) of objects with measurable values. Univariate survival analysis showed that the TNs fractal dimension (*P* = 0.004), TNs number (*P* < 0.001), TNs perimeter sum (*P* = 0.001), TNs cell Delaunay area sum (*P* = 0.007), and stromal cell structure parameters (*P* = 0.011) were negatively correlated with 8-DFS. TNs area average (*P* = 0.011), TNs area variance (*P* = 0.014), and TNs cell nuclei area average (*P* = 0.004) were positively associated with 8-DFS. The TNs cell nuclei area variance (*P* = 0.056) and TNs cell nuclei eccentricity maximum (*P* = 0.077) had no statistically significant correlation with 8-DFS (Table 2).

Semantic-level parameters refer to the information on specific relationships among histological structures, such as area ratio and cell density. Univariate analysis showed that TNs cell nuclei/TNs area ratio (*P* = 0.006), and TNs cell density (*P* < 0.001) were negatively correlated with 8-DFS. TNs area/perimeter ratio (*P* = 0.032) and stromal non-round cell density (*P* = 0.008) were positively associated with 8-DFS (Table 3).

### Screening for image features by 8-DFS prediction

**Figure 3:**
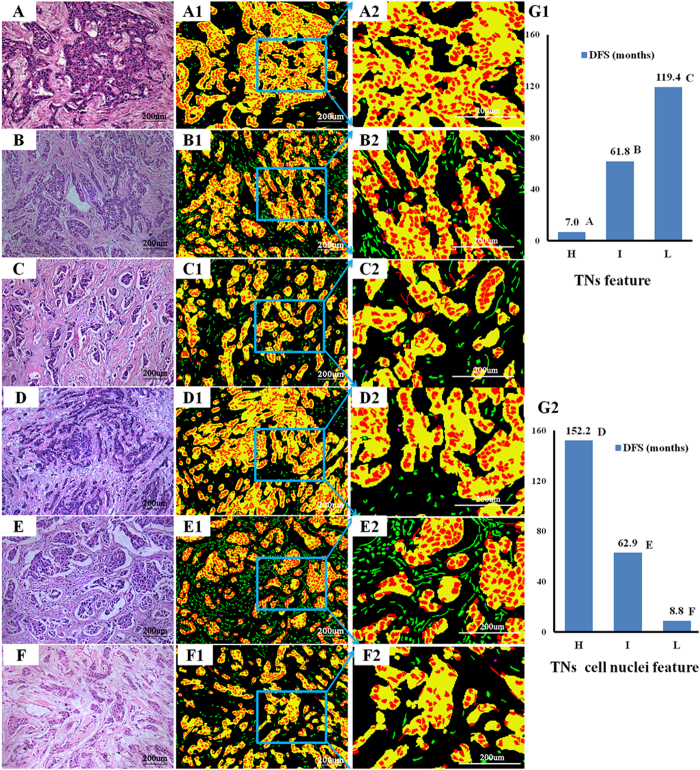
Examples of survival associated TNs feature and TNs cell nuclei feature extracted from IDC HE histopathological images. Of the histologic grade 2 from the original HE images, TNs feature categories had significant independent negative impact on DFS, high TNs feature (panel A, DFS = 7.0 months), intermediate TNs feature (panel B, DFS = 61.8 months), and low TNs feature (panel C, DFS = 119.4 months). Likewise, TNs cell nuclei feature had significant positive impact on DFS, high score (panel D, DFS = 152.2 months), intermediate TNs score (panel E, DFS = 62.9 months), and low score (panel F, DFS = 8.8 months). The second column images are segmentation results in the form of pseudocolor image. The third column images are corresponding local amplification of blue rectangle region in the second column images. The TNs feature (G1) and TNs nuclei feature (G2) and the corresponding DFS of each case respectively. TNs: tumor nests, H: high, I: intermediate, L: low, DFS: disease-free survival.

The above 12 significant object-level and semantic-level parameters were subject to principal component analysis for further reduction, producing two major sets of image features: TNs feature to quantify tumor nests and TNs cell nuclei features to quantify cancer cell nuclei (Table S3 and S4). Univariate survival analysis showed that TNs feature was negatively correlated with 8-DFS (*P* < 0.001), and TNs cell nuclei feature was positively associated with 8-DFS (*P* = 0.021) (Table 3). Examples of correlation between 8-DFS with TNs feature and TNs cell nuclei feature are shown in Fig. 3.

### Validation of image features by multivariate analysis

To validate the clinical significance of the newly selected image features, we carried out a multivariate Cox analysis integrating both traditional and newly identified variables. To avoid the possible multicollinearity among the variables in regression model, a spearman rank correlation test was conducted, and the result showed that no significant correlation among the image features and histologic grade. Then, we integrated traditional prognostic factors including T, N, histologic grade, ER and HER2, and 7 image features that screened by 8-DFS prediction into a Cox proportional hazards model. This resulted in 6 independent prognostic factors. In addition to 2 traditional factors including histological grade and HER2, 4 image features including TNs feature, TNs cell nuclei feature, TNs cell density, and stromal cell structure feature were new independent prognostic factors. Among the 4 image features, TNs cell nuclei feature was a positive prognostic factor, and the other 3 image features were negative prognostic factors for 8-DFS (Table 4).

**Figure 4:**
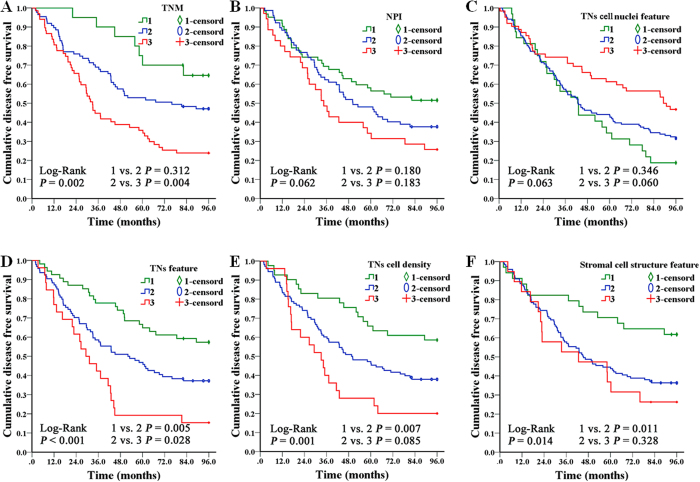
Kaplan-Meier survival curves of TNM, NPI and the 4 independent prognostic image features on 174 histologic grade 2 IDC cases. The results showed that TNM (A), TNs feature (D), TNs cell density (E), and Stromal cell structure feature (F) had significant associations with 8-DFS and gave a better discrimination in histologic grade 2 subgroup. While NPI (B) and TNs cell nuclei feature (C) had no significant correlations with 8-DFS. Furthermore, TNs feature (D), TNs cell density (E) and Stromal cell structure feature (F) could distinguish low risk patients in this group.

**Figure 5:**
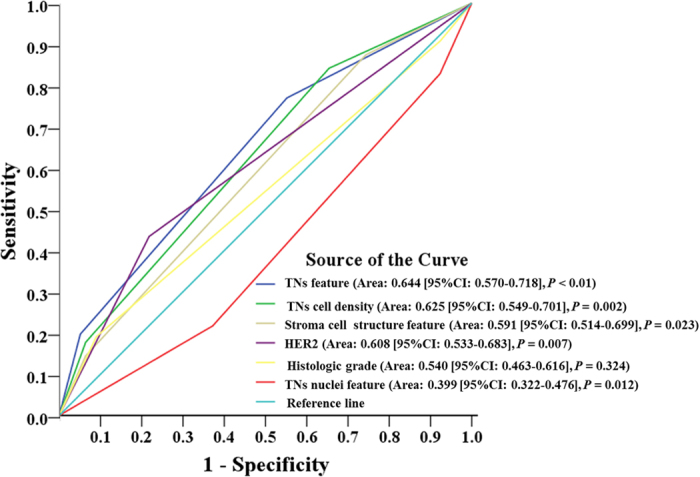
ROC analysis of the predictive performance of those independent prognostic factors for 8-DFS. Among the 6 factors, the area under the curve of the TNs features was the largest one. The TNs features could have better prognostic performance in IDC patients.

To assess the additional value of independent prognostic image features, we related them with histological grade. Result showed that TNs feature, TNs cell density, and stroma cell structure feature gave a better discrimination in histologic grade 2, and could identify low risk patients in this subgroup (Fig. 4). Furthermore, the predictive performance of independent prognostic factors was quantified by the area under the curve (AUC) from a ROC analysis separately. TNs feature could better predict the clinical outcomes of IDC patients compared to other factors (AUC: 0.644 [95%CI: 0.570 - 0.718], *P* < 0.001) (Fig. 5).

## Discussion

CAI could be an important tool to help pathologists in BC prognosis assessment and histological grade^20^,^26^,^27^. Using CAI in this study, we extracted 730 morphological parameters; and 12 parameters, including 6 tumor nests (TNs) parameters, 2 TNs cell parameters, 2 stroma cell parameters, and 2 nuclei parameters were significantly associated with 8-DFS. Four image features, including TNs feature, TNs cell nuclei feature, TNs cell density, and stromal cell structure feature were identified by multivariate Cox analysis to be new independent prognostic predictors. Moreover, they can quantify the microstructure of tissue in HE histopathology images, and yield high reproducible and adequate information.

NGS is demonstrated to have prognostic significance in small tumor groups^28^, but low reproducibility limits its application in breast cancer management^13^. Researchers try to address the issues arising from manual grading by adopting CAI. Several works have proposed solutions for nuclei pleomorphism scoring^21^, tubular formation scoring^29^,^30^ or mitosis detection^31^. The segmentation of nuclei in breast cancer histopathology images has many different applications include extraction of prognostic features, automatic nuclear pleomorphism grading as part of a computer-aided grading system^21^. Along with nucleus pleomorphism, the degree of structural differentiation of the tissue is one of the earliest prognostic factors for BC^29^,^30^. Cancer disrupts the ability of the cells to communicate with each other and organize themselves into structures such as tubules. Approaches attempt at combining the three criteria of NGS to provide a complete automatic grading system^32^,^33^.

The abovementioned works are based on the NGS frame, whereas comprehensive analysis of quantitative morphological features could help identify novel prognostic characteristics from histopathology images. Such work by Tambasco *et al*^34^ used fractal dimension to quantify the complexity of whole epithelial architecture in IHC images from invasive breast cancer. In our work, we extracted a large number of morphometric and topological features in relatively more complex HE histopathology images from IDC. The TNs cell nuclei features generated in this work could quantify nuclei pleomorphism, and the TNs features could objectively measure the morphologic complexity of malignant epithelial architecture. Results showed that both of them were independent prognostic predictors. The TNs feature even had better performance in predicting clinical outcomes than histologic grade and HER2 status in ROC analysis. This suggests that TNs feature and TNs cell nuclei feature could be candidate prognostic factors for IDC.

Furthermore, CAI could help to explore prognostic value of the tumor microenvironment morphologic characteristics for IDC. The NGS examines only three morphological features of epithelial cells, which have failed to accurately classify patients. Our results show that stroma cell structure feature, TNs feature, and TNs cell density which gave a better discrimination in histological grade 2, could further identify low risk patients in this subgroup. Stroma cells such as CAFs, AVCs and IICs are important components of tumor microenvironment, and play important roles in cancer progression^14^. For instance, Beck *et al*^35^ identified three unrecognized stroma features by a C-Path system which was significantly associated with BC survival. Moreover, researchers have used image analysis as complementary to genomic profiling data to quantify cellular heterogeneity in BC^15^ or to discover biomarkers for triple negative breast cancer^16^. In addition to cancer cell features, our work also classified stroma cells into two categories, stromal round cells (IICs) and stromal non-round cells (CAFs and AVCs) according to cell size and the nuclei shape. The stromal non-round cell structure feature was found to be an independent prognostic factor, which positively correlated with 8-DFS. This suggests that analyzing stroma morphologic features may offer significant help to improve prognosis prediction, in consistent with a similar report by Beck *et al*^35^.

However, contrary to the current knowledge, stroma non-round cell density had a positive correlation with survival. Moreover, features such as stroma round cell density and stroma cell nuclei features showed non-significant correlation with 8-DFS. Those findings should not be taken to mean that they have no effect on prognosis. We point out that accurate segmentation of objects of interest is the first step toward automatic image analysis, and this can greatly affect the significance of extracted features. Though we have performed preprocessing, many images remain intractable to the algorithm, due to the heterogeneity of the disease and the strong noise in HE images. In addition, developing computer-aided prognosis for BC based on HE histopathology images is still in the exploratory stage. A major hindrance is that researchers have performed analyses on different size images (WSI or ROIs) and magnification levels (100×, 200×, or 400×) with various segmentation algorithms^36^ and focused on diverse features of objects of interest. Thus, a direct comparison of different methods is not feasible.

This study has several limitations. First, the generalizability of the SVM classifier and marker-controlled watershed algorithm should be validated on another independent dataset. Second, the selection of an optimal image size, magnification, and processing time must be investigated to understand their effects on clinical significance. Third, the prognostic values of the independent predictors need to be validated in prospective study. More work will involve the exploration of intelligent methods like multi-field-of-view^32^ for combination of various features to make full use of the underlying, invaluable, image information. Furthermore, image features alone rarely gives adequate information for prognosis^37^; clinical pathological information and molecular assay data must also be taken into consideration^38^.

In conclusion, it is an urgent and important clinical task to predict future biological behaviors of BC based on the new information extracted from the local tumor itself in addition to conventional pathological features. CAI could be a powerful tool to help extract a huge amount of new information beyond manual analysis, and TNs feature, TNs cell nuclei feature, TNs cell density, and stromal cell structure feature could be new prognostic factors for IDC.

## Materials and Methods

### Patients and tissue slides

This study included 230 patients diagnosed with IDC and treated with intent-to-cure surgery at our hospital. Major treatment information included radical mastectomy (n = 43), modified radical mastectomy (n = 156), and simple mastectomy or breast conserving surgery (n = 31) in terms of surgical treatment; then followed by less intensive chemotherapy (cyclophosphamide + methotrexate + fluorouracil, n = 96), or anthracycline/taxane-based (n = 134) chemotherapy. And radiotherapy was added to patients with over 3 axillary lymph nodes involvement. For patients with HER2 positive status, molecular targeting therapy with Transtuzumab (i.e. Herceptin a monoclonal humanized anti-HER2 antibody) was recommended but not mandatory. Endocrine therapy for with either tamoxifen or third-generation aromatase inhibitors was delivered based on the ER status and clinical guidelines. The tissue slides and formalin-fixed paraffin-embedded (FFPE) tissue blocks, clinical pathological information, and follow-up information of these patients were all available. The study protocol was approved by the Institutional Ethics Committee of Zhongnan Hospital of Wuhan University, and informed consent was obtained from the patients before operation to use tissue samples for scientific researches.

Two expert pathologists (GF Yang, JP Yuan) examined the archived HE slides, selected FFPE tissue blocks for all cases and made new tissue slides for HE and IHC staining (ER, PR, and HER2). Histologic grade for each case was obtained by routine manual analysis of HE images with NGS. The ER and HER2 status of IHC images were evaluated by the above-mentioned expert pathologists. The ER and PR status were defined as the percentage of immunoreactive cells with an intra-nuclear staining of any intensity. The intra-nuclear staining of at least 1% of the cells was interpreted as a receptor-positive result^39^. The HER2 status analysis was performed according to the American Society of Clinical Oncology/CAP guidelines^40^.

### Image acquisition

The digital images were acquired under an Olympus BX52 microscope equipped with an Olympus DP72 camera (Olympus Optical Co., Ltd., Tokyo, Japan) by CRi Nuance multispectral imaging systems (Cambridge Research & Instrumentation, Inc., Woburn, MA, USA) with the help of an expert pathologist (JP Yuan). First, regions of interests (ROIs), the distinct invasive cancer area in images were selected at 100×. ROIs did not contain regions of necrosis, ductal carcinoma *in situ* or improper staining artifacts. Second, in each ROI, only fields containing both tumor nests and stroma were captured at 200×. Finally, to minimize image selection bias, five images per slide were randomly selected from the ROIs images. As a result, 1,150 images were captured under the unified image acquisition parameters and saved in tagged image file format with resolution of 1360 × 1024 pixels.

### Image processing

In order to extract image features related to prognosis, we need to automatic identify and segment histological structures by image analysis methods at first. We applied an image processing pipeline by the following steps: preprocessing, segmentation, postprocessing and feature extraction^24^. First, three preprocessing methods were applied to enhance image quality. Median-filter with a 3*3 kernel was used to de-noise and smooth image; contrast stretching was used to automatically optimize the image contrast; and color normalization was applied to remove color variance and scale batch effects. Second, we used two-step segmentation algorithms to segment objects in images. (1) For nuclei segmentation, color deconvolution was used to extract the hematoxylin color component. Series of mathematical morphology operators were used to remove irrelevant “noisy” structures that may hamper the segmentation for obtaining the nuclei mask. Then the regional minima of the nuclei mask were used to mark candidate nuclei locations. Watershed regions were grown from the markers, after which spurious regions were removed based on shape, texture and boundary saliency. (2) For TNs-stroma segmentation, the pixel-level color features via the local homogeneity model and texture features of the pixel via the fast algorithm^41^ were used. Then an SVM classifier was trained with randomly selected labeled pixels by the abovementioned expert pathologists, and the images were segmented with the trained SVM classifier. Third, as the segment methods lack robustness to noise and cannot classify all the pixels to the objects accurately. Expert-pathologist aided judgments were conducted to eliminate the incorrect segmentations in the postprocessing step. In the final feature extraction step, multiple levels (i.e. pixel-level, object-level, and semantic-level) of morphological features^18^ were extracted from different objects.

### Statistical analyses

The image features we generated from image analysis are continuous variables. In order to classify patients into different risk subgroup, we converted continuous variables into categorical variables before performing statistical analyses by using the X-tile software^42^. Then univariate survival analysis and principal component analysis were used for features dimensionality reduction. Kaplan-Meier methods were used to identify 8-year disease free survival (8-DFS) associated features, and significance among subgroups was calculated by log-rank test. The 8-DFS was defined from the date of surgery to the date of BC-specific recurrence/distant metastasis or date of last follow-up. Multivariate Cox proportional hazards regression model was performed to identify new independent prognostic factors from 8-DFS associated features. Two sided *P* < 0.05 was considered as statistically significant. Receiver operating characteristic (ROC) curve analysis was used to determine predictive value of the independent prognostic factors. All statistical analyses were performed with SPSS version 19.0 (SPSS Institute, Chicago, IL, USA).

## Additional Information

**How to cite this article**: Chen, J.-M. *et al.* New breast cancer prognostic factors identified by computer-aided image analysis of HE stained histopathology images. *Sci. Rep.* **5**, 10690; doi: 10.1038/srep10690 (2015).

## Supplementary Material

Supporting Information

## Figures and Tables

**Figure 1:**
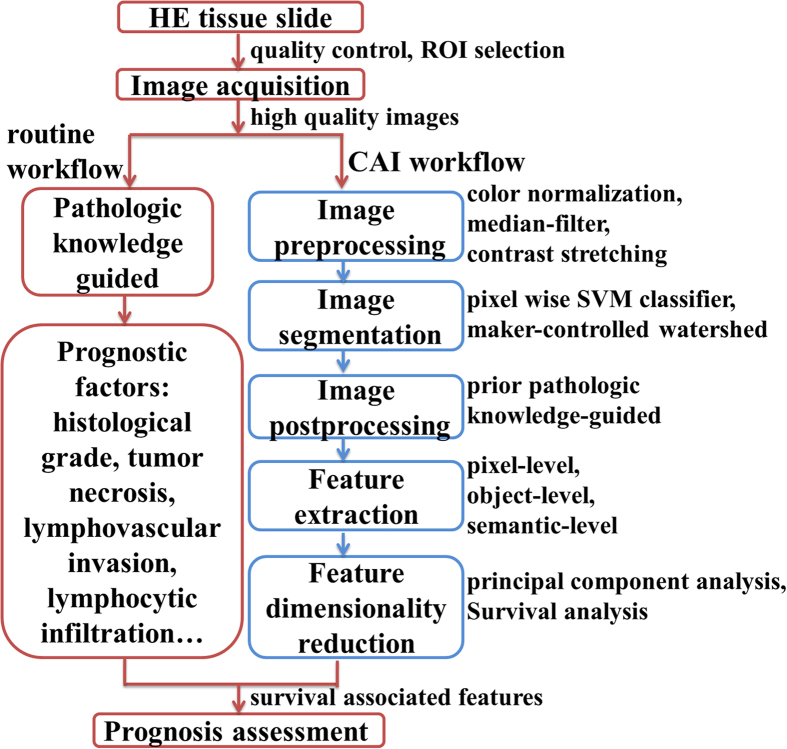
The main steps of computer-aided image analysis (CAI) proposed in this study (blue/right frame) in comparison with traditional histopathology-based prognosis assessment (red/left frame). Image preprocessing was performed to ensure high-quality data are processed. Image segmentation, postprocessing, and feature extraction converted image information into quantitative features. Feature dimensionality reduction was used to explore survival associated features. ROI: the regions of interest, SVM: support vector machine.

**Table 1:** Major demographic, clinical and pathological characteristics of 230 IDC patients.

**Characteristics**	**Value (%)**	**Recurrence, n (%)**	**Median 8-DFS (month)**	**8-DFS Rate (%)**	***P*value**
Age (M ± SD, yr)	53.5 ± 11.6	N/A	N/A	N/A	N/A
Tumor size (cm)					0.777
1 (≤2.0)	64 (27.8)	43 (67.2)	53.0	32.8	
2 (2.0 - 5.0)	133 (57.8)	86 (64.7)	50.1	35.3	
3 (≥5.0)	33 (14.4)	23 (69.7)	42.9	30.3	
Lymph node status§					0.011
0 (0)	77 (33.5)	45 (58.4)	82.0	41.6	
1 (1 - 3)	66 (28.7)	44 (66.7)	53.0	33.3	
2 (4 - 9)	56 (24.3)	38 (67.9)	50.1	32.1	
3 (≥10)	31 (13.5)	25 (80.6)	25.8	19.4	
Histologic grade#					0.054
1	20 (8.7)	14 (70.0)	76.8	30.0	
2	174 (75.6)	109 (62.6)	55.9	37.4	
3	36 (15.7)	29 (80.6)	36.6	19.4	
ER					0.082
0 (negative)	125 (54.3)	86 (68.8)	42.9	31.2	
1 (positive)	105 (45.7)	66 (62.9)	62.0	37.1	
HER2					<0.001
0 (negative)	147(63.9)	86 (58.5)	77.7	41.5	
1 (positive)	83 (36.1)	66 (79.5)	32.9	20.5	
TNM stage					0.001
1 (I)	27 (11.7)	15 (55.6)	92.9	44.4	
2 (II)	113 (49.2)	68 (60.2)	52.1	39.8	
3 (III)	90 (39.1)	69 (76.7)	32.9	23.3	
NPI*					0.007
1 (≤2.8)	73 (31.7)	40 (54.8)	87.3	45.2	
2 (2.8 - 4.4)	108 (47.0)	75 (69.4)	49.9	30.6	
3 (>4.4)	49 (21.3)	37 (75.5)	33.1	24.5	

N/A: not applicable; DFS: disease free survival; M: mean; SD: standard deviation; ER: estrogen receptor; HER2: human epidermal growth factor receptor 2; TNM: tumor-node-metastasis.

^§^The number of positive axillary lymph nodes;

^#^Histologic grade: Nottingham grading system, NGS^12^;

^*^NPI = tumor maximum invasive cancer size in centimeters × 0.2 + lymph node (LN) stage (1, 2, or 3) + histologic grade (1, 2, or 3)^7^.

**Table 2:** Analyses of object-level morphological features regarding 8-DFS.

**Variables**	**Recurrence, n (%)**	**Median 8-DFS (month)**	**8-DFS Rate (%)**	***P* value**
TNs fractal dimension				0.004
1 (<1.60)	29 (54.7)	90.8	45.3	
2 (1.60 - 1.70)	87 (65.9)	52.1	34.1	
3 (>1.70)	36 (80.0)	26.0	20.0	
TNs number				<0.001
1 (<52.0)	42 (50.6)	90.8	49.4	
2 (52.0 - 80.4)	85 (72.0)	42.9	28.0	
3 (>80.4)	25 (86.2)	29.3	13.8	
TNs perimeter sum				0.001
1 (<25076.04)	30 (51.7)	90.8	48.3	
2 (25076.04 - 33151.54)	79 (66.4)	49.9	33.6	
3 (>33151.54)	43 (81.1)	29.6	18.9	
TNs cell Delaunay area sum				0.007
1 (<1241981.0)	47 (58.0)	82.2	42.0	
2(1241981.0 - 1263738.0)	43 (63.2)	45.1	36.8	
3 (>1263738.0)	62 (76.5)	34.5	23.5	
Stromal cell structure feature				0.011
1 (<26.21)	19 (48.7)	96.0	51.3	
2 (26.21 - 32.57)	111 (67.7)	45.3	32.3	
3 (>32.57)	22 (81.5)	22.9	18.5	
TNs area average				0.011
1 (<9543.71)	89 (73.6)	40.3	26.4	
2 (9543.71 - 15615.19)	42 (61.8)	67.2	38.2	
3 (>15615.19)	21 (51.2)	90.8	48.8	
TNs area variance				0.014
1 (<30965.25)	101 (73.7)	42.2	26.3	
2 (30965.25 - 51733.13)	28 (57.1)	78.6	42.9	
3 (>51733.13)	23 (52.3)	89.2	47.7	
TNs cell nuclei area average				0.04
1 (<190.67)	46 (82.1)	42.7	17.9	
2 (190.67 - 213.68)	74 (66.7)	42.9	33.3	
3 (>213.68)	32 (50.8)	92.9	49.2	
TNs cell nuclei area variance				0.056
1 (<72.80)	21 (84.0)	39.0	16.0	
2 (72.80 - 97.75)	100 (65.8)	45.3	34.2	
3 (>97.75)	31 (58.5)	87.5	41.5	
TNs cell nuclei area eccentricity maximum				0.077
1 (<0.98579)	16 (57.1)	87.3	42.9	
2 (0.98579 - 0.99323)	114 (65.9)	52.1	34.1	
3 (>0.99323)	22 (75.9)	34.5	24.1	

TNs: Cancer cell group with various geometrical and morphological features is called tumor nests (TNs).

**Table 3:** Analyses of semantic-level morphological features regarding 8-DFS.

**Variables**	**Recurrence, n (%)**	**Median 8-DFS (month)**	**8-DFS Rate (%)**	***P* value**
TNs cell nuclei area/TNs area ratio				0.006
1 (<0.206)	13 (46.4)	96.0	53.6	
2 (0.206 - 0.320)	113 (66.5)	50.3	33.5	
3 (>0.320)	26 (81.2)	26.6	18.8	
TNs cell density				<0.001
1 (<0.0011)	24 (47.1)	96.0	52.9	
2 (0.0011 - 0.0016)	101 (68.7)	49.9	31.3	
3 (>0.0016)	27 (84.4)	26.6	15.6	
TNs area/perimeter ratio				0.032
1 (<13.22)	40 (76.9)	39.0	23.1	
2 (13.22 - 19.17)	60 (69.0)	43.2	31.0	
3 (>19.20)	52 (57.1)	82.2	42.9	
Stromal non round cell density				0.008
1 (<0.00030)	28 (82.4)	22.9	17.6	
2 (0.00030 - 0.00061)	104 (65.8)	49.9	34.2	
3 (>0.00061)	20 (52.6)	89.2	47.4	
TNs feature*				<0.001
1 (< –1.28)	35 (50.0)	90.8	50.0	
2 (–1.28 - –0.09)	87 (69.0)	43.2	31.0	
3 (> - –0.09)	30 (88.2)	31.2	11.8	
TNs cell nuclei feature#				0.021
1 (<1.60)	26 (81.2)	42.7	18.8	
2 (1.60 - 2.94)	93 (68.4)	42.9	31.6	
3 (>2.94)	33 (53.2)	89.2	46.8	

^*^TNs feature = 0.260 × TNs number + 0.107 × TNs perimeter sum-0.281 × TNs area average-0.272 × TNs area variance-0.268 × TNs area/perimeter ratio;

^#^TNs cell nuclei feature = 0.048 × TNs cell nuclei eccentricity maximum + 0.482 × TNs cell nuclei area average + 0.478 × TNs cell nuclei area variance + 0.246 × TNs cell nuclei area/TNs area ratio.

**Table 4:** Multivariate Cox proportional hazards model to predict 8-DFS in 230 IDC patients.

**Variables**	**Coefficient**	**Hazard Ratio (95% CI)**	***P* value**
T	0.082	1.086 (0.843, 1.399)	0.524
N	0.120	1.128 (0.947, 1.343)	0.176
Histologic grade	0.387	1.472 (1.051, 2062)	**0.025**
ER	–0.276	0.759 (0.543, 1.061)	**0.107**
HER2	0.658	1.931 (1.368, 2.725)	**<0.001**
TNs fractal dimension	0.161	1.174 (0.830, 1.661)	0.364
TNs feature	0.283	1.327 (1.001, 1.759)	**0.049**
TNs cell Delaunay area sum	0.182	1.200 (0.921, 1.564)	0.177
TNs cell density	0.486	1.625 (1.177, 2.244)	**0.003**
TNs cell nuclei feature	–0.317	0.729 (0.537, 0.989)	**0.042**
Stroma cell structure feature	0.467	1.596 (1.142, 2.229)	**0.006**

TNs: tumor nest; HER2: human epidermal growth factor 2; T: tumor; N: node; ER: estrogen receptor.
