# Supplementary material for: New breast cancer prognostic factors identified by computer-aided image analysis of HE stained histopathology images
Source: Sci Rep. 2015 May 29;5:10690. doi: 10.1038/srep10690 (PMC4448264; doi:10.1038/srep10690)
Supplement: Supporting Information [file srep10690-s1.pdf]

New breast cancer prognostic factors identified by computer-aided image analysis of HE stained histopathology images

Jia-Mei Chen [1], Ai-Ping Qu [2], Lin-Wei Wang [1], Jing-Ping Yuan [1], Fang Yang [1], Qing-Ming Xiang [1],

Ninu Maskey [3], Gui-Fang Yang [3], Juan Liu [2,] *, Yan Li [1,] *

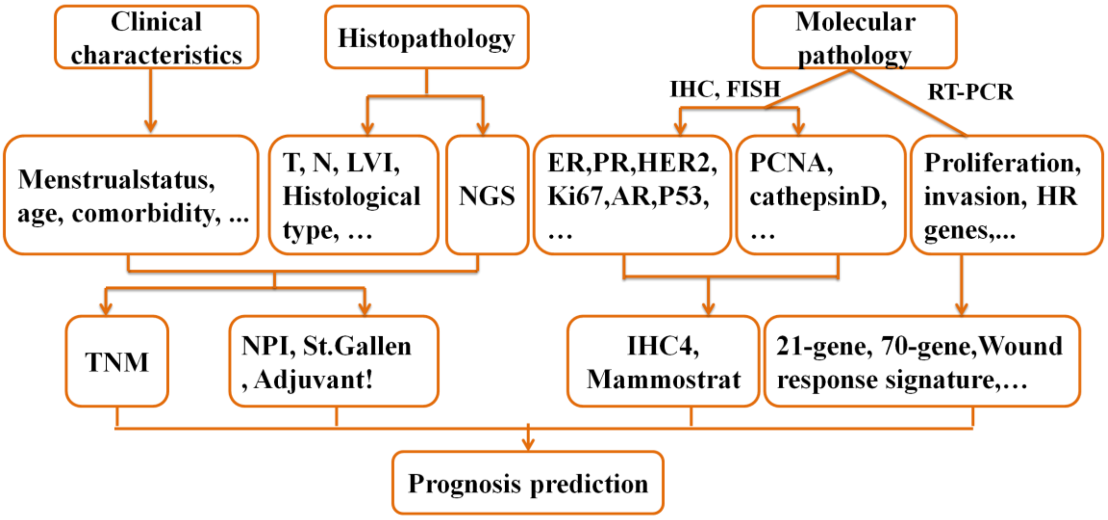

Figure S1 | Overview of breast cancer prognostic prediction tools in current clinical study. These tools generally integrated information on clinical characteristics, gross pathology, histopathology and molecular pathology. Abbreviations: IHC (immunohistochemistry), FISH (fluorescence in situ hybridization), RT-PCR (reverse transcription-polymerase chain reaction), T (tumor size), N (lymph node status), LVI (lymph-vascular space invasion), NGS (Nottingham grading system), ER (estrogen receptor), PR (progesterone receptor), HER2 (human epidermal growth factor receptor 2), HR (hormone receptor), AR (androgen receptor), PCNA (proliferating cell nuclear antigen), NPI (Nottingham Prognosis Index), IHC4 (ER, PR, HER2, and Ki-67), Mammostrat (p53, HTF9C, CEACAM5, NDRG1 and SLC7A5).

Table S2. Multiple level image features extracted from HE histopathology images

Feature level	Features	Feature number
Pixel-level features		400
Intensity	smoothness, third moment, consistency	6
Color	average and standard deviation of the gray-scale image and the RGB color image components	6
Texture	LBP, Haralick, LBPHF, GLRM, GLCM	388
Object-level features		314
Morphometry	average, standard deviation, maximum and minimum	214

	of each image object area, perimeter, size, eccentricity, solidity, circularity, convex area, the major and minor axis of the ellipse, fractal dimensions	
Topological	Voronoi diagrams, Delaunay triangulation, Minimum spanning tree, and the Ripleys K function of cell nuclei	100
Semantic-level features	nest area to stroma area ratio, area-perimeter ratio of tumor nests and stroma, nuclei- cytoplasm ratio, tumor nests cell density, stroma round cell density, stroma non round cell density, nest cell nuclei to stroma cell nuclei area ratio	16

LBP: local binary patterns; LBPH: the rotation invariant local binary patterns with histogram Fourier transform; GLRM: gray-level run-length matrix; GLCM: gray-level co-occurrence matrix

Table S3. Total variance explained of principal component analysis.

Component	Initial eigenvalues			Extraction sums of squared loadings			KMO
	Total	% of Variance	Cumulative %	Total	% of Variance	Cumulative %	
			TNs feature				
Z1	3.288	65.756	65.756	3.288	65.756	65.756	0.778
Z2	0.945	18.901	84.657				
Z3	0.425	8.494	93.151				
Z4	0.253	5.062	98.213				
Z5	0.089	1.787	100.000				
			TNs cell nuclei feature				
Z1	1.911	47.771	47.771	1.911	47.771	47.771	0.539
Z2	0.997	24.923	72.694				
Z3	0.881	22.035	94.729				
Z4	0.211	5.271	100.000				

TNs: cancer cell group with various geometrical and morphological features is called tumor nests (TNs); KMO: Kaiser-Meyer-Olkin measure of sampling adequacy.

Table S4. The component score coefficient matrix of principal component analysis.

Features	Component Z1

TNs feature*	
TNs number	0.260
TNs perimeter sum	0.107
TNs area average	-0.281
TNs area variance	-0.272
TNs area/perimeter ratio	0.268
TNs cell nuclei feature#	
TNs cell nuclei area eccentricity maximum	0.048
TNs cell nuclei area average	0.482
TNs cell nuclei area variance	0.478
TNs cell nuclei area/TNs area ratio	0.246

*TNs feature = 0.260 × TNs number + 0.107 × TNs perimeter sum - 0.281 × area average - 0.272 × TNs area variance - 0.268 × TNs area/perimeter ratio; #TNs cell nuclei feature = 0.048 × TNs cell nuclei eccentricity maximum + 0.482 × TNs cell nuclei area average + 0.478 × TNs cell nuclei area variance + 0.246 × TNs cell nuclei area/TNs area ratio.
